# Mucosal Epithelial Jak Kinases in Health and Diseases

**DOI:** 10.1155/2021/6618924

**Published:** 2021-03-16

**Authors:** Narendra Kumar, Longxiang Kuang, Ryan Villa, Priyam Kumar, Jayshree Mishra

**Affiliations:** ^1^Department of Pharmaceutical Sciences, ILR College of Pharmacy, Texas A&M Health Science Center, Kingsville TX 78363, USA; ^2^Academy High School, Santa Gertrudis ISD, Kingsville, TX 78363, USA

## Abstract

Janus kinases (Jaks) are a family of nonreceptor tyrosine kinase that include four different members, *viz*., Jak1, Jak2, Jak3, and Tyk2. Jaks play critical roles in immune cells functions; however, recent studies suggest they also play essential roles in nonimmune cell physiology. This review highlights the significance of epithelial Jaks in understanding the molecular basis of some of the diseases through regulation of epithelial-mesenchymal transition, cell survival, cell growth, development, and differentiation. Growth factors and cytokines produced by the cells of hematopoietic origin use Jak kinases for signal transduction in both immune and nonimmune cells. Among Jaks, Jak3 is widely expressed in both immune cells and in intestinal epithelial cells (IECs) of both humans and mice. Mutations that abrogate Jak3 functions cause an autosomal severe combined immunodeficiency disease (SCID) while activating Jak3 mutations lead to the development of hematologic and epithelial cancers. A selective Jak3 inhibitor CP-690550 (Xeljanz) approved by the FDA for certain chronic inflammatory conditions demonstrates immunosuppressive activity in rheumatoid arthritis, psoriasis, and organ transplant rejection. Here, we also focus on the consequences of Jak3-directed drugs on adverse effects in light of recent discoveries in mucosal epithelial functions of Jak3 with some information on other Jaks. Lastly, we brief on structural implications of Jak3 domains beyond the immune cells. As information about the roles of Jak3 in gastrointestinal functions and associated diseases are only just emerging, in the review, we summarize its implications in gastrointestinal wound repair, inflammatory bowel disease, obesity-associated metabolic syndrome, and epithelial cancers. Lastly, we shed lights on identifying potential novel targets in developing therapeutic interventions of diseases associated with dysfunctional IEC.

## 1. Introduction

The nonreceptor tyrosine kinases are intracellular tyrosine kinases where the family of Janus kinases (Jaks) include four members: Jak1, Jak2, Jak3, and Tyk2. Conventionally, they were thought to be essential for cytokine receptor-mediated signaling during immune responses. However, over a decade of recent studies indicate that they are also involved in mediating signal transduction for growth and proliferation in nonhematopoietic cells and other tissues. Among the four members of Jak family, loss of Jak1 in mice shows perinatal lethality in the newborn mice which exhibit significant reduction in thymocytes and B-lymphocytes thereby indicating that Jak1 is indispensable for overall survival [1]. Jak2 deficiency has been correlated with the human malignancies such as myeloid metaplasia with myelofibrosis characterized by excess proliferation of the cells of one or more of myeloid lineages [2]. Tyk2 is another member of Jak family where loss of Tyk2 in mice shows that though they are viable but their IL-12 signaling, which is important for host defense, is compromised. Additionally, these mice also show enhanced Th2 responses and impaired proinflammatory immune activities [3]. Human Jak3 deficiency is primarily characterized by phenotypes of SCID [4]. Though relatively less severe, such phenotypes are also observed in experimental Jak3-KO mice where deficiency in B cell and NK cells was observed. Historically, Jak3 has been reported to play critical role in many cellular pathways in lymphoid cells. However, over the past decade of studies indicate essential roles of Jak3 in nonlymphoid cells including in mucosal epithelial functions. In human, mutations in Jak3 related severe combined immunodeficiency disease feature reduction of immune cells such as T cells and NK cells. A lack of function in B cells was also observed in patients with mutated Jak3 and present with deficiency in IgG and IgM. On the other hand, an abnormal activation of Jak3 results in hematologic and epithelial malignancies. Moreover, studies showed that Jak3 is also expressed in other organs, including intestine of both humans and mice [5, 6]. This combined with the observance of epithelial malignancy [7] due to constitutive activation of Jak3 led to several investigations into nonhematopoietic functions of Jak3, and later on Jak3, it was reported to be not only expressed in gastrointestinal epithelial cells but also in the regulation of several gastrointestinal mucosal functions [8]. Reports suggest that expression of Jak3 was decreased in IECs of human obese patients compared to healthy subjects [9]. In this study, colonic tissue sections from normal or obese human subjects' immunostained with Jak3 antibody showed that Jak3 is expressed in the luminal mucosal surfaces of healthy human subjects; however, in obese subjects, Jak3 expression is significantly decreased. Moreover, obesity-associated decrease in colonic expression of Jak3 was observed in both male and female subjects compared with their normal counterparts. Another study suggested that Jak3 activator IL-2 facilitated differentiation in human-derived intestinal organoid culture [10] implicating not only expression but also the functional role of Jak3 in human. Moreover, this study also shows a clear differentiation of crypt-like and villus-like structures in a 3D multicellular intestinal organoid culture which was developed using either human pluripotent stem cells or adult intestinal stem cells. These organoids show all four major cell types of the small intestinal epithelium thereby recapitulating the architecture and cellular diversity of the intestinal epithelium and making them an excellent in vitro model system for studying Jak3 functions in human intestinal development and diseases [10]. Extending these, study also showed that Jak3 expression specifically in mouse IECs plays key role in several intestinal physiological processes [1, 11] where Jak3 is not only expressed in colonic mucosa but absence of Jak3 led to decreased expression of the differentiation markers for enterocytes including villin and carbonic anhydrase as confirmed through western analysis.

The gastrointestinal (GI) epithelium in human body has several important roles such as providing a physical barrier against infections, protecting the body from the harsh environment of the gut lumen, and making chemical barrier through production of mucus, which forms a protective gel-like layer over the epithelial surface [12]. The GI epithelium also makes a surface that facilitates selective absorption of water and nutrients from ingested foods. In addition, the GI epithelial forms a barrier between the luminal contents and the host immune system which are due to a delicate balance between different cellular processes including of cell proliferation, differentiation, and apoptosis. This balance is achieved through among others the various signaling pathways that play essential roles in regulating aforementioned epithelial functions during normal development and maintaining epithelial homeostasis in gastrointestinal tract. This is because deregulation of some of these signaling pathways is associated with intestinal diseases such as IBD, IBS, and intestinal neoplasia [13]. Among various biomolecules, protein kinases play a key role in regulating these signaling pathways thereby regulating several intestinal functions including the development and regeneration of the intestinal epithelium [14].

Intestinal restitution is a process through which mucosal wounds are rapidly repaired and is among the important epithelial functions mediated by the cells of gastrointestinal mucosa. Defects in intestinal restitution are associated with several chronic inflammatory diseases including leaky-gut syndrome, ulcerative colitis, and Crohn's disease. A delicate balance between pro- and anti-inflammatory mediators orchestrates this process through cross talk between neutrophils, macrophages, and the epithelium to promote wound repair by a coordinated process of epithelial-mesenchymal transition, cell migration, proliferation, mesenchymal-epithelial transition, and differentiation of IECs. An imbalance of the intestinal cytokine milieu results in delayed wound repair as seen in several chronic inflammatory diseases. Therefore, an improved understanding of repair mediators is important for not only designing better therapeutic agents but also promoting healing of intestinal wounds as an instigator of several chronic inflammatory diseases.

Apoptosis is another important epithelial function responsible for maintenance of the cellular homeostasis through balancing proliferation and cellular death in the GI tract. Dysregulation of apoptosis is seen in several pathological conditions in the GI tract. Among the cytokines, tumor necrosis factor (TNF) particularly TNF-*α* plays an important role in inducing apoptosis in the cells of the GI tract where it has been shown that during crypt-villus migration of the cells, the apoptosis and detachment of enterocytes are induced at the villus tip by the activation of caspase activities [15].

It has been reported that chronic inflammatory diseases such as irritable bowel syndrome (IBS), inflammatory bowel disease (IBD), functional bowel disorders, and celiac disease are the manifestation of increased permeability of GI tract. Therefore, maintenance of intestinal barrier functions is essential to mitigating these chronic inflammatory conditions. Among kinases, Jak3-deficient mice have been reported to display the defective barrier functions which are linked to mislocalization of the adherent junction proteins [11].

Among the factors that contribute to intestinal barrier functions, IECs have robust mucosal detoxification system. Drug transporters are a part of this system that reduces the xenotoxicity caused by the therapeutic drugs, environmental pollutants, carcinogens, and natural toxins. Xenotoxic damage to GI tract causes different inflammatory triggers through the disruption of IEC integrity. This leads to formation of inflammatory lesions, which are found during active inflammation particularly in the patients with IBD. In a healthy individual, an appropriate response to such a disruption is required to rapidly reseal the lesion through mucosal healing through a stepwise process of cell migration, cell proliferation, and differentiation in order to restore barrier functions. Alternatively, in individuals predisposed to IBD, an active inflammation arises due to compromise in intestinal wound repair. These conditions have been associated with a significant decrease in the expression of ABC family of drug transporters in the IECs. These decreased expressions lead to reduced capacity of the IECs to detoxify the luminal xenobiotic challenge and are thought to interfere with the efficiency of regenerative responses.

In this review, we focus on the unique roles mediated by Jak family members with emphasis on Jak3. We emphasize on the epithelial functions of Jak3 by elucidating the mechanistic roles in mucosal wound repair, homeostasis, epithelial barrier functions, mucosal detoxification, and epithelial-mesenchymal transition (EMT). Finally, we illustrate Jak3's contribution in mucosal development of gastrointestinal tissue and prevention and amelioration of chronic inflammatory conditions and shed lights on the correlation between mechanistic understanding and development of novel therapeutic interventions.

## 2. Epithelial Functions of Jak3

### 2.1. Mucosal Wound Healing

**Figure 1:**
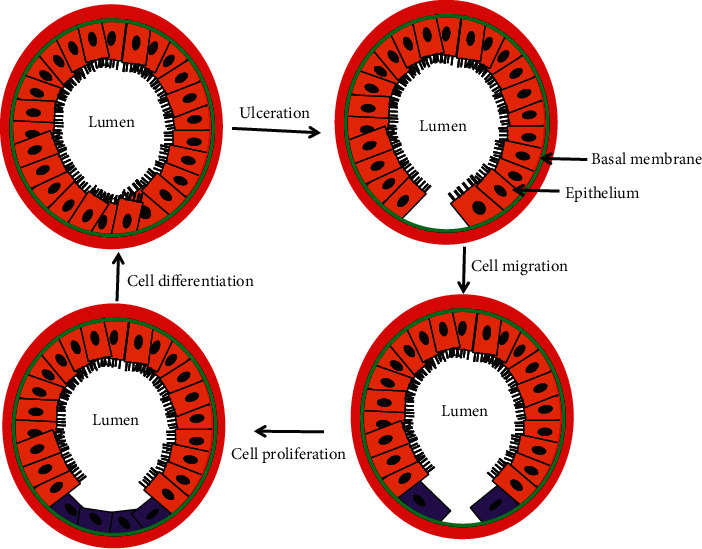
Schematic diagram for the cellular processes involved in intestinal restitution.

**Figure 2:**
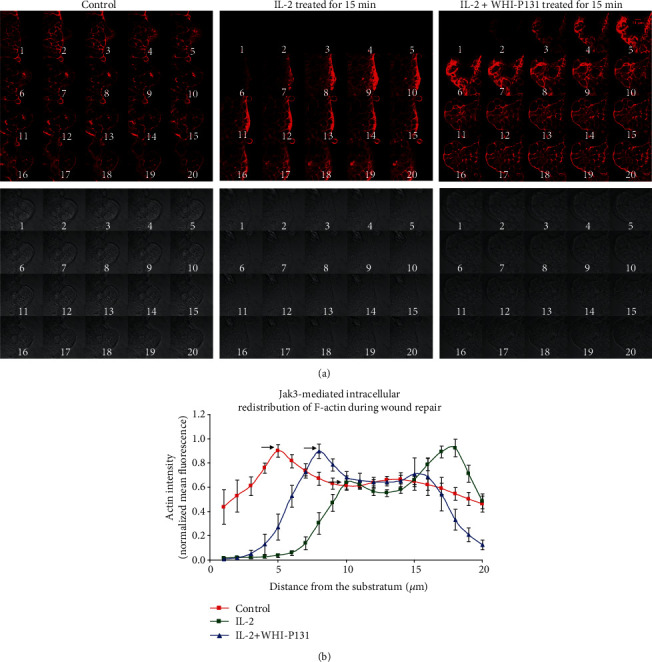
IL-2-stimulated Jak3 activation leads to disassembly of actin filaments from the basal plane of migrating cells. (a) Planar distribution of f-actin in HT-29 Cl-19A cells at the wound edge of control cells; cells treated with IL-2; and cells treated with IL-2+WHI-P131. The lower images in each panel are the corresponding bright field images. (b) Normalized f-actin intensity for each plane as a function of the distance from the substratum [19].

Intestinal epithelial cells (IEC) respond to the mucosal injury through stepwise process of cell spreading and migration to facilitate covering of the wound, followed by cell proliferation to replace the damaged cells and cellular differentiation upon would closure to restore the functional integrity of the mucosal surface (Figure 1). At the cellular level, immune cells located underneath the mucosal epithelial lining specifically, the lymphocytes secrete several cytokines, which have profound impact on the surviving cells surrounding the wound. Among the cytokines, interleukin 2 (IL-2) is a pleotropic cytokine that promotes several epithelial functions. This is because functional IL-2 receptors are endogenously expressed not only in several human IEC lines such as Caco-2, HT-29, and T-84 cells, but also in primary human IECs thereby implying a physiological role for IL-2 in regulating epithelial functions [16, 17]. IL-2 modulates growth and differentiation of immunocytes during inflammatory response and maintains mucosal integrity through promoting repair of intestinal epithelial mucosa [18, 19]. Additionally, while IL-2-deficient mice develop progressive inflammatory bowel disease closely resembling human ulcerative colitis (UC) [20], UC patients exhibit a significant reduction in the expression of IL-2 [21]. These results suggest that IL-2 plays an essential role in regulating intestinal structure and mucosal immunity [10]. Moreover, the formation of 3D structures of organized crypt and villus domains through coculture where human T-lymphocytes induced the maturation of intestinal organoids (hIOs) from human pluripotent stem cell further indicated a role of IL-2. In this study, T cell-derived IL-2 was identified as an activator of STAT3 as the major factor inducing this maturation. These hIOs exposed to IL-2 closely mimic the adult intestinal epithelium and showed comparable expressions of mature intestinal markers and intestine-specific functional activities even upon in vivo engraftment [10]. These indicate IL-2's role in proliferation and differentiation of IEC cells [22, 23]. To demonstrate the mechanism, the human-derived differentiated cell culture model suggests that IL-2 promotes wound repair of IEC in a dose-dependent manner [24]. Specifically, the cells treated with low doses of IL-2 had significantly increased wound repair than the control where higher concentration of IL-2 had opposite effects on wound repair. Mechanistically, IL-2 activates Jak3 and the activated Jak3 phosphorylates intestinal epithelial-specific cytoskeletal protein villin, and the Jak3-villin complex promotes actin reorganization through actin-severing activity by Jak3-phosphorylated villin. These severing activities generate new actin nuclei which in turn facilitate actin polymerization and bundling at the wound edge. Together, these actin reorganizing activities promote epithelial motility for the enterocyte through cellular filopodial and lamellipodial extension (Figures 2(a) and 2(b)). Inversely, an inhibition of Jak3 prevented IL-2-induced villin phosphorylation and inhibited wound healing [19].

### 2.2. Role of Jak3 in Mucosal Homeostasis

Epithelial homeostasis in intestinal mucosa is maintained by a strict equilibrium between cell proliferation in the crypt, crypt-villus migration of these cells, maturation and differentiation of these cells as they migrate, and apoptotic shedding of the aged cell from the villus tip. In the large and small bowel, aged, differentiated enterocytes are removed constantly and replaced by new cells originated by undifferentiated intestinal stem cells at the crypt [25]. Through a strict balance in these stepwise processes, the epithelial layer maintains the intestinal barrier. Failure to achieve and/or maintain this equilibrium has negative consequences for several chronic inflammatory conditions with both intestinal and systemic ramifications. Evidence suggests that apoptotic signaling may also promote inflammatory processes by releasing chemokines loaded extracellular vesicles with a potential to recruit and activate immune cells [26]. Dysregulation in mucosal apoptosis as indicated by excessive cell shedding has been noticed and suggested to be responsible for compromised barrier and pathogenesis of human inflammatory bowel disease (IBD) such as Crohn's disease (CD) and ulcerative colitis (UC). Excessive cell shedding and loss of barrier are also predictor of remission and disease relapse in IBD [27]. Like cell migration during wound repair, IL-2 also stimulates cell proliferation in a dose-dependent manner from 10 to 50 U/ml. However, a higher dose causes a decrease in cell number. This is further justified where the biopsy samples in normal individuals showed a concentration range of 0 to 9 U/ml of IL-2 [28]. However, patients with IBD had a concentration between 0 and 20 U/ml. Though IBD patients also report a higher concentration of lamina propria mononuclear cells, the main source of IL-2 in intestines, these cells had less IL-2 receptors in these patients. On the other hand, patients with CD showed a decrease in peripheral blood mononuclear cells, and those with UC showed a higher IL-2 concentration compared to CD. This finding may encourage the use of cyclosporin A (CSA), an IL-2 inhibitor, in immunosuppressive patients with UC. Indeed, these patients treated with CSA achieved significant improvement compared to placebo. This suggests that a high concentration of IL-2 induces apoptosis, which interrupts homeostasis. CSA reduces IL-2 concentration, which prevents mucosal apoptosis. Since patients with CD already had a lower concentration of IL-2, CSA was not a beneficial for these conditions. There are also some inconsistent in vivo studies reporting that IBD patients and controls showing higher or lower concentration of IL-2. Such inconsistencies are due to factors such as the concentration of activated lymphocytes, level of IL-2 receptor expression on the surface of activated lymphocytes, and soluble IL-2 receptor concentration.

**Figure 3:**
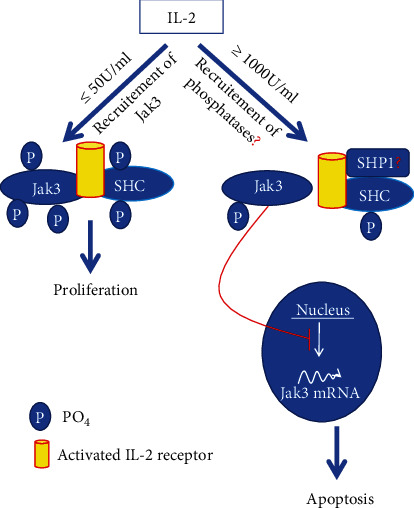
Proposed model of IL-2-induced intestinal epithelial homeostasis [24].

Mechanistically, IL-2 induces activation of Jak3 tyrosine phosphorylation, and the tyrosine-phosphorylated Jak3 interacts with p52ShcA (Shc) where these interactions facilitate mucosal homeostasis. At lower IL-2, Jak3 interactions with Shc promote proliferation and cell growth suggestively by inducing Ras activation. Jak3 interactions with Shc also act as regulators of Jak3 dephosphorylation through direct interactions of Shc with both Jak3 and tyrosine phosphatases (Figure 3). At a higher concentration, IL-2 promotes dephosphorylation of both Shc and Jak3 by activating the Src homology region 2 domain-containing phosphatase-1 (SHP1). This leads to loss of interactions between Jak3 and Shc and Jak3 redistribution to the nucleus instead of the functional localization at the cell membrane where an increased nuclear localization of Jak3 was observed in apoptotic cells. Apart from Jak3, Jak1 and Jak2 are also reported to be present in the nucleus of certain cells often under conditions of high rates of cell growth. Nuclear Jaks have now been shown to affect gene expression by activating other transcription factors besides the STATs and exerting epigenetic actions. For example, Jak phosphorylation of histone H3 represses gene expression and is implicated in leukemogenesis [29]. Transcriptional studies indicated that though the transcription of Shc-mRNA was increased in IECs under both low and high doses of IL-2, high doses of IL-2 decreased the expression of Jak3 mRNA resulting in apoptosis. It is also observed that in the absence of either Shc or Jak3 genes, there is increased apoptosis resulting from an oversensitivity IECs to IL-2. These were proved using multiple approaches including pharmacological inhibition and specific targeting of Jak3 by shRNA. The knockdown of Jak3 expression caused apoptosis in IEC in the presence of IL-2. In this study, the Jak3 shRNA used was a tetracycline-regulated system of Jak3 shRNA expression where lentiviral-mediated transduction of pTRIPZ-sh-Jak3 was done. pTRIPZ-sh-Jak3 also contained red fluorescence protein (RFP) under a doxycycline-regulated promoter for visualization in live cells. The expression of Jak3 shRNA in the transduced cells was induced by supplementing doxycycline in the growth media, further confirming the doxycycline regulation of Jak3 shRNA expression and the downregulation of Jak3 protein. Furthermore, there was no effect of the lentivirus carrying Jak3 shRNA alone or in combination with doxycycline on proliferation in the absence of IL-2. However, when Jak3 was knocked down by doxycycline-regulated expression of Jak3 shRNA, IL-2 induced loss of cells at low concentrations. These along with other studies conclusively proved the absolute requirement of Jak3 in IEC homeostasis, particularly during IL-2 signaling [10, 11, 24]. A reduced IL-2 may also indicate susceptibility to colorectal cancer. For example, in IBD immunocompromised patients, a reduced/lack of IL-2 activity could impair immune surveillance by antineoplastic lymphokine-activated killer or natural killer cells and may result in a higher risk for IBD-associated colorectal cancer.

The transmolecular mechanism of the regulation of IL-2-stimulated Jak3 activation by Shc indicates that Jak3 autophosphorylation was the rate-limiting step during Jak3 transphosphorylation of Shc where Jak3 directly phosphorylated two tyrosine residues in Src homology 2 (SH2) domain and one tyrosine residue each in calponin homology 1 (CH1) domain and phosphotyrosine interaction domain (PID) of Shc. Jak3 phosphorylates Tyr420 and Tyr458 in the SH2 domain of Shc. The other two specific tyrosine residues in CH1 and PID domains phosphorylated by Jak3 remain to be determined. The structural determinants that regulate the interactions between Jak3 and Shc show a direct interaction between FERM domain of Jak3 with the CH1 and PID domains of Shc. These not only characterize Jak3 interaction with Shc, but also demonstrate the molecular basis of intracellular regulation of Jak3 activation in mucosal epithelial cells where Jak3 interactions with Shc acted as regulators of Jak3 dephosphorylation through direct interactions of Shc with both Jak3 and tyrosine phosphatases.

Adapter functions of Shc depend on various factors such as phosphorylation. Shc relays both intracellular and extracellular signals. The activation of Shc signaling is associated with poor prognosis in cancer patients. Constitutive activation of Jak3 has been linked to various cancers. However, data suggests that Jak3 can still be constitutively active without mutation in the Jak3 protein itself but through an impairment of its interactions with Shc. This is because Shc recruited tyrosine phosphatases SHP2 and PTP1B to Jak3 and thereby dephosphorylated Jak3. Such impairment can be caused by mutations in any of the proteins such as Shc or phosphatases which dephosphorylate Jak3, i.e., SHP2 or PTP1B where dephosphorylation facilitates in dissociation.

### 2.3. Role of Jak3 in Mucosal Differentiation

**Figure 4:**
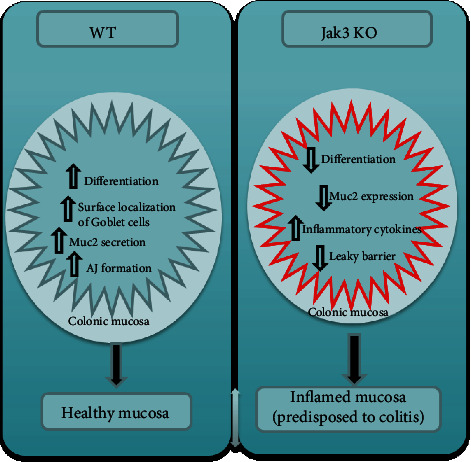
Proposed model for the role of Jak3 in colonic mucosal health and predisposition to colitis [11].

Differentiation is the process through which the cells from the proliferative compartments (crypt) of the intestinal mucosal lining migrate to the villus and at the same time change into specialized cell types. As gastrointestinal epithelium is a complex system with several functions, in response to extracellular communication, the crypt located progenitor cells differentiate into one of the four major cell types, viz., enterocytes with absorptive and immunoglobulin secretory functions, enteroendocrine cells with hormone secretory functions, Paneth cells with antimicrobial defense function, and goblet cells with mucin secretory function. These cells undergo continuous renewal, and through their diverse functions, they work in concert to maintain intestinal physiology and promote host defense. Among different cell types, enterocytes make up the most abundant cell types followed by goblet and enteroendocrine cells which constitute ∼5% and ∼1% of the total epithelial cells, respectively. Among the transcription factors, Cdx2 plays a central role where it was reported that ectopic expression of Cdx2 gene induces intestinal epithelial cell differentiation [30]. Though several signaling pathways play important roles in patterning gut development and in regulating epithelial differentiation, defects in any of them are linked to many intestinal disorders including frequent intestinal infection, IBD, and celiac disease. Jak3 plays an important role in such a scenario because Jak3 not only promotes expression of colonocyte differentiation markers in human IEC but loss of Jak3 expression in genetically modified mouse leads to compromised differentiation and predisposition of colitis. Defining Jak3's functions in human IEC shows that knockdown of its expression leads to diminished expression of the differentiation markers for enterocytic lineages including villin and carbonic anhydrase. In addition to enterocytes, the intestinal mucosal of both human and mice is covered with a thick layer of heavily glycosylated protein mucin that forms mucus layer. This layer provides a physical barrier between gut microbes and mucosal epithelium where decreased thickening of mucous layer is associated with increased severity of colitis in both humans and mice [31]. Both missense mutations in the muc2 gene or total lack of intestinal mucin (muc2 KO) in mouse leads to mucosal friability, spontaneous wound formation in mucosa, and development of colitis [32]. It is known that Jak3 also plays an essential role in the formation of colonic mucus layer where a loss of its expression in Jak3-KO mouse leads to compromise in goblet cell differentiation. As revealed by the intestinal mucosa of these mice, there is a decrease in surface localization of goblet cells toward the colonic lumen (Figure 4) resulting in discontinuous mucous layer phenotype and decreased expression of secretory mucin, muc2 in these mice [11]. To dissect intestinal epithelial cell-specific and immune cell-specific roles of Jak3, additional studies using flox-Jak3 mated either with Vil1Cre mouse (Jak3f/f/Vil1-cre) or with VavCre mouse (Jak3f/f/VavCre) were done. The results show that both recapitulate the global knockout conditions indicating that both the tissue-specific Jak3 play essential role in protection from disease severity. Results show both groups had increased severity towards DSS-induced colitis in IEC-Jak3^−/−^ and IMM-Jak3^−/−^ compared to their littermate controls. Additionally, both of these mice show increased disease activity scores for colitis as reflected by shortened colon length, hepatic steatosis with increased liver to body weight ratio, and decreased crypt heights compared to their littermate controls. The tissue-specific role of Jak3 also shows association between ulcerative colitis and hepatic inflammation in both IEC-Jak3-KO and Imm-Jak3-KO groups indicating essential roles of both IEC-specific and immune cell-specific Jak3 in maintaining tissue homeostasis and immunotolerance. Additionally, immunohistochemical analyses also indicated Kupffer cell activation-associated hepatic inflammation (unpublished data, manuscript under review).

Jak3 is also reported to facilitate the differentiation of colonic mucosa through additional mechanisms involving *β*-catenin-mediated adherens junction (AJ) formation where Jak3 activation promotes *β*-catenin phosphorylation at Tyr654 which in turn allows *β*-catenin to interact with E-cadherin. These interactions act as an essential step in the formation of AJ. This was further confirmed by multiple strategies. First, using Jak3-KO mouse showed decreased localization of *β*-catenin at the AJ thereby interrupting the barrier functions of the IEC. Second, using pharmacological inhibition of Jak3 resulted in similar outcome in terms of *β*-catenin localization at the AJ. Third, AJ localization of *β*-catenin was determined in 2-week postconfluent IEC model where the cells were transduced with lentiviruses having doxycycline-regulated expression of RFP-tagged Jak3 shRNA. Results from this doxycycline regulated expression of Jak3 shRNA indicated an intact AJ in the IEC as reflected by continuous expression of *β*-catenin around the cellular periphery only in those cells which were transduced with lentiviruses containing RFP-tagged Jak3 shRNA but with no doxycycline. Since doxycycline activated the expression of Jak3 shRNA, indeed, the doxycycline supplemented IEC cells showed expression of RFP-tagged Jak3 shRNA and a substantial loss of *β*-catenin expression at AJ where doxycycline alone had no effects on either expression or localization of *β*-catenin [11]. Additionally, it is postulated that interactions between Jak3 and *β*-catenin could also translocate Jak3 to the nucleus, influencing the expression of other differentiation markers for intestinal mucosa [11].

### 2.4. Jak3 and Mucosal Barrier Function

The intestinal epithelial barrier composed of a single layer of columnar epithelial cells lining in the gut that contains four types of epithelial cells. Laid over the epithelial is a layer of mucus forming critical barrier that provides limitation of the exposure of epithelial cells to the gut microbiome. The chemical barrier of the mucosal layer along with the lamina propria resident immune cell, the epithelial cell layer, performs a critical role as the first physical barrier against external factors including symbiotic relationship with gut microbiome [33].

The human intestine is in constant contact with over 10^14^ commensal bacteria from over 500 different species. These bacteria not only confer health benefits through dietary digestion and gut immunity but also prevent colonization of pathogens. To maintain intestinal barrier, mucosal epithelium is posed with a daunting task of distinguishing commensal bacteria from enteric pathogens [34]. Enteric pathogens need to breach the glycocalyx, mucins, IgA, and antimicrobial peptide defense to come to close contact with epithelial cells that line the intestine. Toll-like receptors (TLRs) and Nod-like receptors (NLRs) present on IEC control mucosal immunity through sensing not only microbial-derived ligands but also self-ligands. For example, TLR2 and TLR4 sense fragments of the extracellular matrix component hyaluronan [35] and the Hsp70 protein, respectively [36] apart from microbial components. TLR-mediated activation of nuclear factor kappa-light-chain-enhancer of activated B cells (NF-*κ*B) is involved in the production of inflammatory cytokines including IL-6, IL-1*β*, MCP-1, and TNF-*α*.

**Figure 5:**
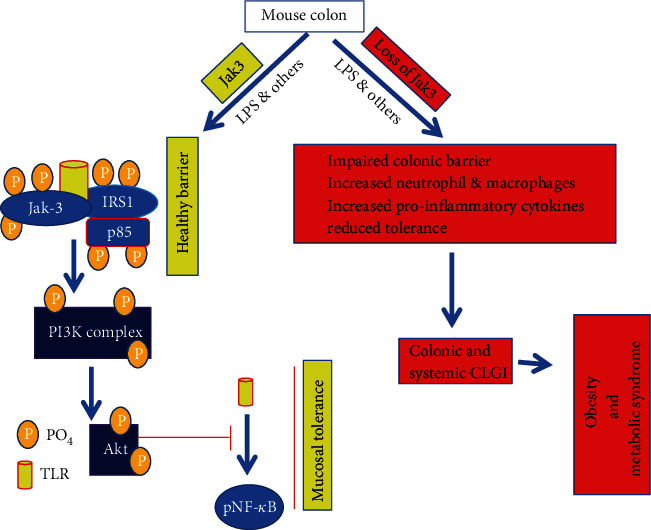
Proposed models for Jak3-mediated mucosal tolerance and predisposition to obesity and MetS [1].

Study suggests key role of Jak3 in reduced expression of TLR2 and TLR4 in mucosal epithelial cells. Mechanistically, stimulation of TLR in both human and mouse intestinal epithelium is sufficient for intracellular activation of Jak3, which leads to subsequent activation of PI3K-Akt axis through Jak3 interaction with p85, the regulatory subunit of PI3K. These interactions are mediated by the tyrosine-phosphorylated adapter protein IRS1 that in turn is essential for reduced TLR expression and NF-*κ*B activation. These results were reconfirmed in vivo using Jak3-KO mouse where increased expression and activation of TLR2 and TLR4 were observed (Figure 5). Alternatively, increased lipopolysaccharide (LPS) sensitization is responsible for enhanced TLR expression resulting in compromised tolerance of intestinal mucosa. Additionally, sensitization by LPS also leads to posttranscriptional regulation of TLR4 mRNA where LPS facilitates binding of human antigen R (HuR) proteins to the 3′-UTR of TLR4 mRNA. These bindings in turn control TLR4 level via TLR4 mRNA degradation. On the other hand, enhanced activation of either TLR2 or TLR4 leads to increased phosphorylation of NF-*κ*B in the colon. As Jak3 is known to phosphorylate HuR which in turn regulates HuR binding to TLR4-mRNA, this provides additional mechanism of TLR suppression by Jak3 in intestinal mucosa. Yet another machinery involves transcription factor ZNF160 which is expressed in the IEC. ZNF160 is less sensitive to LPS and interacts with scaffold proteins to recruit histone deacetylase at the 5′ region of the TLR4 gene in cells that are less sensitive to LPS compared to the IECs that are more sensitive. Together, these indicate that IECs entail additional controls to suppress TLR expression where Jak3 could be pivotal in such suppression. This is because loss of Jak3 expression leads to failure of colonic barrier, which is responsible for increased levels of proinflammatory cytokines in the intestinal tissue. These increases in turn culminate into colonic and systemic CLGI. Thus, regulation of Jak3 expression plays an indispensable role towards prevention of CLGI associated with several health complications **(**Figure 5**)**.

### 2.5. Jak3 in Epithelial-Mesenchymal Transition (EMT)

**Figure 6:**
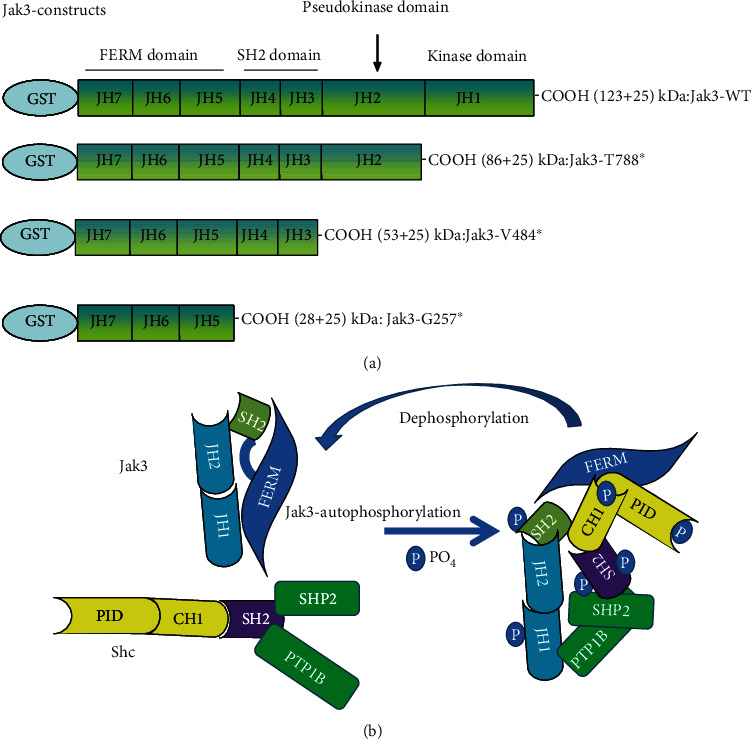
Adapter protein Shc regulates Jak3 activation through regulating Jak3 interactions with tyrosine phosphatases [40]. (a) Schematic representation of GST-Jak3-WT and mutants [39]. (b) Model for Shc-mediated Jak3 dephosphorylation [40].

**Figure 7:**
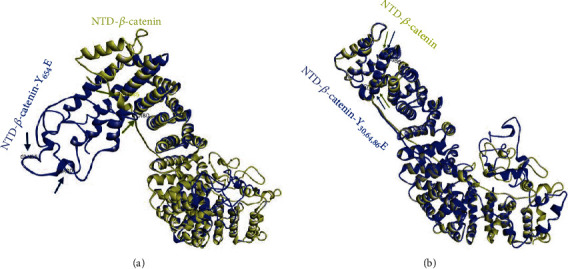
Molecular dynamics of Jak3-mediated phosphorylation sites in *β*-catenin [8]. Arrows (gold, *β*-catenin–WT; blue, *β*-catenin—Y654E) indicate the corresponding positions of Ala80, Gly85, and Val166 in the modeled proteins. Note that superimposed *β*-catenin–WT (gold) on Y30E, Y64E, and Y86E (blue) in (b) shows overlap of the positional markers Gly85 and Val166 in the structure of the protein, indicating a close resemblance in conformation of the NTD of *β*-catenin–WT and *β*-catenin–Y30E, Y64E, and Y86E and a reversal of orientation from *β*-catenin–Y654E in (a).

In the context of mucosal wound repair, epithelial-mesenchymal transition (EMT) is a key biological process through which the differentiated polarized epithelial cells surrounding the wound gets dedifferentiated through multiple biochemical changes to attain a mesenchymal cell phenotype with increased migratory capacity. During wound repair, termination of EMT through reinforcement of adherens junction (AJ) is necessary after successful completion of the proliferation step (Figure 1). A subtle balance between growth factors and cytokine coordinates EMT. An AJ is a multimolecular protein complex that plays a key role in maintaining the differentiated and polarized state of mucosal epithelial tissue architecture. AJ is created by the interactions between among others, sticky homotypic transmembrane protein cadherin. Cadherins in turn interact with catenin family of proteins which are linked intracellularly with a thick actin filament-based cortical structures. *β*-Catenin is a member of catenin family that facilitates multiple functions in the cells including formation of AJ by creating a bridge between E-cadherin and *α*-catenin [37] and activating Wnt signaling through transcription factor activity [38]. Jak3 is reported to play a role in tyrosine phosphorylation-mediated regulation of EMT by *β*-catenin in IECs. Report suggests that Jak3 phosphorylates *β*-catenin in a time-dependent manner with a t½ of 34 s [8]. This report shows *β*-catenin as a direct substrate for Jak3 where Jak3 autophosphorylation serves as the rate-limiting step in Jak3-*β*-catenin interactions. Apart from *β*-catenin, Jak3 also phosphorylates actin-binding protein villin [19, 39] and adapter protein p52ShcA [24, 40] where the phosphorylation of p52ShcA by Jak3 precedes the phosphorylation of *β*-catenin and villin. Since the adapter protein p52ShcA also interacts with protein tyrosine phosphatases, this shows that p52ShcA plays a dual role of recruiting Jak3 substrate for phosphorylation and facilitating their dephosphorylation through the recruitment of phosphatases, a cyclic process of phosphorylation and dephosphorylation (Figures 6(a) and 6(b)) [40]. So far, as cytokine-mediated signaling is concerned, in immune cells, Jaks are known to bind their respective cytokine receptors through N-terminal located FERM domain [41]. However, in epithelial cells, FERM domain of Jak3 (Figure 6(a)) not only does intramolecular interactions and activation of Jak3 kinase domain but also binds to SH2 intradomain thereby maintaining a closed conformation in a nonphosphorylated molecule of Jak3 (Figure 6(b)) [39, 42]. Furthermore, NTD domain of *β*-catenin binds to both Jak3-FERM and Jak3 kinase domains where Jak3-SH2 domain partially hinders these bindings [8]. Furthermore, interactions of the NTD domain of *β*-catenin with Jak3 lead to phosphorylation of three tyrosine residues located in the NTD domain, viz., Tyr30, Tyr64, and Tyr86. This study also demonstrated that Jak3 autophosphorylation was the first and rate-limiting step during Jak3 binding to *β*-catenin. Additionally, preceding phosphorylation of *β*-catenin at Tyr654 is essential for its interaction with autophosphorylated Jak3 and additional phosphorylation. Interestingly, both receptor and nonreceptor tyrosine kinase phosphorylate Tyr654 in *β*-catenin and this phosphorylation in *β*-catenin leads to either nuclear localization [43] or breast carcinomas [44] or EMT [45]. However, Jak3-mediated additional phosphorylation at Tyr30, Tyr64, and Tyr86 in *β*-catenin apart from Tyr654 prevents this protein from exiting AJ and reinforcing barrier functions. Elevated tyrosine kinase activity is also reported in other cell types that stabilize *β*-catenin-mediated AJs [46, 47]. A coordination in the regulation of cell-cell adhesion and gene transcription is essential for controlled cell proliferation and differentiation. Jak3-mediated enhanced barrier functions are demonstrated under in vivo conditions using a mouse model where Tyr654-phosphorylated *β*-catenin is localized to AJ of intestinal mucosa in wild-type mouse expressing Jak3. However, loss of Jak3 using genetically modified mouse showed a loss of AJ localization of Tyr654-phosphorylated *β*-catenin, and this loss of AJ localization of partially phosphorylated *β*-catenin is associated with compromised intestinal barrier functions and inflammation. Since Jak3 requires activation by cytokine or epithelial growth factor (EGF) to phosphorylate additional three tyrosine residues in *β*-catenin, it appears that under physiological conditions, a fine balance in the concentrations of Jak3 activators might synchronize EMT and barrier functions through Jak3 interactions with *β*-catenin. On the other hand, mucosal IECs play a critical role in overall human health because of its anatomical location where huge concentration of immune cells beneath mucosal surfaces in lamina propria needs to be kept at bay from the gut microbiome with trillions of microbes towards luminal sides [48]. Under such a hostile environment, regulation of EMT becomes critical not only for IEC-mediated mucosal wound repair during healthy conditions but also for the prevention of the onset of neoplastic transformation followed by metastasis of epithelial cells that could result in colorectal cancer. This is also because it is known that Tyr654-phosphorylated *β*-catenin facilitates cellular proliferation, and Jak3-mediated additional phosphorylation at Tyr30, Tyr64, and Tyr86 in Tyr654-phosphorylated *β*-catenin can be critical in putting a brake on proliferation, particularly after a successful completion of mucosal wound repair followed by transition into redifferentiated state through the formation of AJ and reinforcement of mucosal barrier functions. Jak3-mediated specific phosphorylation at these tyrosine residues in *β*-catenin is also confirmed through phosphomimetic approaches using molecular modeling and comparison of the resulting structure. These molecular structures show that Tyr654 phosphorylation in *β*-catenin induces a significant change in structure that is reversed by further phosphorylation at the NTD-domain located Tyr30, Tyr64, and Tyr86 in *β*-catenin (Figure 7), and this reversed form of *β*-catenin stabilizes AJ through its interactions with AJ located *α*-catenin thereby enhancing mucosal IEC barrier functions. Moreover, because colonic polyps are characterized as a transition state of the progression from normal epithelial cells to cancerous cells where EMT is critical to this progression, Jak3 acts as a critical regulator of uncontrolled epithelial proliferation (and hence the formation of colonic polyps) by suppressing EMT following successful mucosal wound repair and promoting mesenchymal-epithelial transition (MET). Furthermore, since human colonic polyps show sustained expression of the EMT marker N-cadherin [49, 50] and forced expression of N-cadherin in epithelial cells facilitate neoplastic transformation and colonic polyp formation [51], this physiopathological implications indeed indicate that human colonic polyps have significantly reduced Jak3 expression and localization compared with healthy mucosal counterparts and these polyps have a significantly elevated level of the EMT marker N-cadherin [8].

### 2.6. Jak3 in Mucosal Efflux Function

ATP-binding cassette (ABC) family of transmembrane transporters is one of the largest protein super families with diverse functions [52]. Under physiological conditions, these proteins transport lipids, sterols, metabolic wastes, and therapeutic drugs across intracellular and extracellular membranes of the cells. They actively transport the metabolites and utilize the energy from ATP hydrolysis to transport against the solute concentration gradient [53]. They also provide a xenobiotic protective function through pumping a wide variety of compounds out of the cell [54]. There are three different principal efflux proteins in the gastrointestinal (GI) tract.

**Figure 8:**
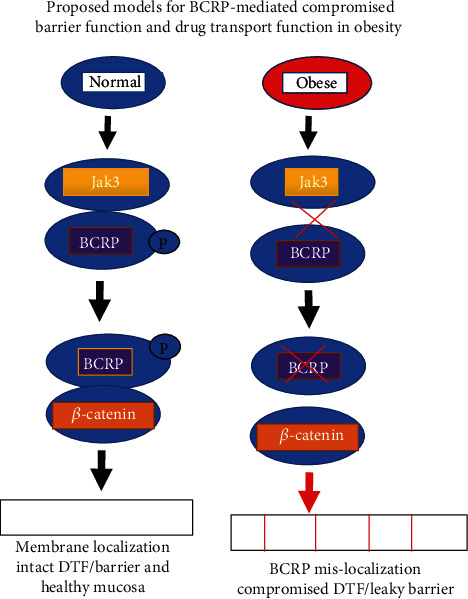
Proposed models for BCRP phosphorylation mediated mucosal barrier function and predisposition to obesity [9].

Breast cancer resistance protein (BCRP) is a member of ABC transporter proteins, and the primary function of this transporter is to eliminate toxic xenobiotic substances in the (GI) and biliary tract. GI tract is the major site for human interaction with xenobiotics where Jak3 regulated BCRP primarily through posttranslational mechanisms. Both obese human and Jak3-KO mice showed decreased BCRP protein expression and altered localization in the mucosal surface of the GI tract. BCRP mRNA expression is generally maximal in the duodenum where colonic expression ranged from 76 to 50% of the duodenum [55]. The regulation of BCRP expression and their functional consequences during obesity indicate no significant differences in colonic BCRP mRNA expression between normal and obese human subjects or mice thereby ruling out the possibility of transcriptional regulation during obesity. However, significant difference in tyrosine phosphorylation of BCRP in normal and obese indicated towards a posttranslational regulation. Both male and female obese human had significantly decreased expression of colonic JAK3, and knockout of JAK3 in mouse led to significant decrease in colonic BCRP expression and decreased colonic drug efflux functions [9]. These indicate a role of JAK3 in regulating BCRP protein expression and functions. Furthermore, tyrosine phosphorylation of BCRP by Jak3 facilitates luminal membrane localization through tyrosine phosphorylation-mediated BCRP interactions with AJ protein *β*-catenin. Jak3 inhibition and knockdown of *β*-catenin both led to loss of membrane localization of BCRP, decrease in BCRP-protein expression, and a significant increase in intracellular accumulation of Rh1234. Study in human tissues also confirmed these where obese subjects had loss of BCRP interactions with *β*-catenin whereas healthy subjects had these interactions intact. Interestingly, loss of these interactions led to increased colonic leakage of inulin. BCRP interactions with *β*-catenin could also regulate AJ where loss of these interactions is associated with colonic leakiness-associated CLGI as seen in obese subjects [56, 57]. It would also be interesting to know diets causing obesity had an impact on the regulatory elements of the genes for Jak3 and/or BCRP. Overall, the molecular basis of compromised intestinal drug efflux during obesity indicated BCRP interactions with Jak3 was essential for BCRP tyrosine phosphorylation (BCRP-P) that facilitated BCRP-P interactions with *β*-catenin (Figure 8). Further, BCRP-P interactions with *β*-catenin facilitated BCRP membrane localization, BCRP protein expression, and drug efflux functions. Currently, we are working towards determining the structure-function correlation of Jak3 and BCRP that would shed light on structural determinant of BCRP responsible for its posttranslational regulation.

## 3. Jak3 and Intestinal Diseases

Intestinal diseases affect any segment of the small intestine or large intestine, encompass both acute and chronic conditions, and cover a wide range of diseases like IBD [58], IBD-associated colon cancer, obesity, obesity-associated metabolic syndrome, obesity-associated colon cancer [59], NK cell enteropathy, and epitheliotropic intestinal T cell lymphoma (EITL). This section mainly focuses on the association between Jak3 signaling and epithelial function in these pathophysiological conditions.

### 3.1. Jak3 in Inflammatory Bowel Disease (IBD)

IBD is an autoimmune disease that includes both Crohn's disease (CD) and ulcerative colitis (UC) [60]. This chronic inflammatory condition of the intestine is characterized elevated levels of the production of proinflammatory cytokines that continuously triggers the intestinal inflammation [61]. Among the signaling pathways responsible for the pathogenesis of IBD, several studies suggest mucosal hypoxia-triggered signaling pathways play a key role where details on the specific roles of hypoxia-inducible factors (HIFs), nuclear factor kappa B (NF-*κ*B), prolyl hydroxylases (PHDs), and possible corresponding therapeutic potentials in treating IBD are known in the literature [62]. Using IECs, the importance of dysregulation in RIPK pathways and corresponding therapeutic approaches for IBD are also reported [63]. A few studies provided important insight into microbiota, epigenetics, and cell death mechanism in NOX2-dependent regulation of IBD [64–66]. The Jak/STAT signaling pathways have been implicated in the pathogenesis (IBD), especially since a Jak inhibitor recently has been shown to be effective in the treatment of UC [67]. Complex cytokine-driven inflammation is reported to efficiently modulate therapeutic inhibition of the Jak proteins [67]. Jaks bind to the intracellular domains of several cytokine receptors. In majority of immune cells, it was reported that this binding leads to phosphorylation of a class of transcription factor called signal transducer and activation of transcription (STAT) involved in transcription of different genes including proinflammatory cytokines. This led to the development of oral Jak inhibitors (JAKi) to selectively target different isoforms of Jak and have anticytokine therapeutic effects in treating IBD. Tofacitinib is an oral Janus kinase (Jak) inhibitor or a pan-Jak inhibitor to be precise that inhibits Jak members, in particular Jak1 and Jak3, achieving a broad limitation of inflammation by interfering with several cytokine receptors [68]. These indicate some of the mechanism-based therapeutic strategies to target Jak-STAT-mediated inflammatory signaling pathway in treating IBD using small molecule drugs. However, many of these small molecule inhibitors are unsuccessful in treating IBD due to severe GI side effects. In addition to the conventional role of Jaks in Jak-STAT pathways in immune cells, research from our laboratory, however, suggested that knocking out *Jak3* gene in mice increases severity toward dextran sulfate sodium-induced colitis. This led us to investigate the role of Jak3 in context of nonimmune cell functions in the intestine. We showed that during DSS-induced colitis, Jak3-KO mice had shorter colon length, smaller cecum length, and shorter crypt heights compared to their littermate controls. To demonstrate the molecular basis of such symptoms, we showed that Jak3 is essential for the expression of differentiation markers in intestine and loss of jak3 gene leads to compromised differentiation through decreased expression of the differentiation markers for both enterocytes and secretory lineages. In addition, Jak3-KO mice also showed the signs of CLGI as reflected by increased basal myeloperoxidase activity in both serum and colonic tissues indicating systemic and localized neutrophilia and increased basal levels of acute and chronic proinflammatory cytokines such as IL-6 and IL-17a in colon [11]. These point to alternative strategies where studies suggest using large molecule biologics such as anti-cytokine antibodies or anti-leukocyte trafficking antibodies including anti-IL-23, anti-TNF, anti-IL-12/-23 dual, to target cytokines, and anti-integrin to prevent immune cell infiltration to gut site of inflammation as strategies in treating IBD [69].

### 3.2. Jak3 in IBD-associated Colon Cancer and Sporadic Colon Cancer

IBD-associated colon cancer is one of most serious complications of IBD where long-term UC patients have an increased risk of developing IBD-associated colorectal (CRC) [70]. This is because IBD results in intestinal damage due to a chronic state of inflammation that leads to the predisposition to colitis-associated CRC in individual with IBD. Though the molecular basis of the IBD-related carcinogenesis is yet to be thoroughly understood, it is speculated that IBD-associated cancers occur because of the chronic inflammatory state in the intestine which attracts tumor-promoting lymphocytes into colon which in turn produce growth-promoting cytokines for the cancer cells [71, 72]. In addition to IBD-associated CRC, the molecular events that lead to cancer development in patients with UC are also parallel to those occurring in the cases of sporadic colon cancer. Both involve a multistep process of clonal evolution characterized by genetic instability, clonal expansion, the progressive accumulation of genetic abnormalities, and the development of dysplasia and carcinoma. For example, abnormalities of the tumor suppressor gene p53, APC, and Rb have similar frequencies in UC and sporadic colon cancer. In the colon, tumor growth is significantly influenced by the relative balance between proliferation and apoptosis; Jak3 plays an important role in keeping the balance between proliferation and apoptosis. Syncope et al. studied the proliferative and apoptotic indexes during the adenoma-carcinoma sequence of human colonic tumor samples and found a decrease in apoptosis during tumor progression. Indeed, a low apoptotic index is associated with a poor cancer prognosis.

Chemoprevention of colorectal cancer with long-term sulfasalazine and 5-ASA treatment has been demonstrated in patients with UC. Treatment with 5-ASA is associated with an increase in apoptosis and a decrease in proliferation of colorectal mucosa and has been shown to lower the rate of spontaneous mutation. The cellular basis of the anticancer effect of these agents, although not fully elucidated, seems to involve induction of apoptosis to clear damaged cells and inhibition of proliferation through a combination of mechanisms.

The majority of cases of colon cancer are sporadic, but a distinct marker is a somatic mutation of the adenomatous polyposis coli (APC), which appears to be a determinant early step in the development of colorectal cancer. Functional Jak3 is expressed in human intestinal enterocytes including colon cancer cells. Jak3 plays an essential role in epithelial cell migration in response to IL-2, and a correlation can be made to metastatic potential of colon cancer cells. The inhibition of Jak3 activation has resulted in the loss of tyrosine phosphorylation of villin and a significant decrease in cell migration of the intestinal epithelial cells. Increased levels of autoantibodies to villin were identified in the serum of colon cancer patients. It appears that villin expression deregulated in cancerous colon cells resulting in leaking to the serum. Therefore, deregulation of Jak3 expression has been linked to colon cancer. The transcription factor STAT3 is often constitutively activated in cancerous cells. Jak3 tyrosine phosphorylates SH2 domain of the STAT3, resulting in its activation. Jak3 inhibitors inhibit the phosphorylation of STAT3, which introduce cell-cycle arrest and apoptosis. Our own observation through examination of the histological sections of 22 different tumors from patients with stage II or stage IV colon cancer shows that both STAT3 and Jak3 proteins are not only expressed in these tumors but their activated forms are also frequently present in these tumor tissues. These results are further corroborated using qRT-PCR which shows the presence of Jak3 and STAT3 mRNAs in of all these colon cancer cells demonstrating the significance of uncontrolled activation of Jak3 in neoplastic transformation (unpublished data).

### 3.3. Jak3 in Obesity-Associated Metabolic Syndrome

Intestinal inflammation is considered as an early event in obesity and insulin resistance [57, 73] whereas animal model suggests that consumption of high-fat-diet leads to the development of low-grade intestinal inflammation **[**1**]**. The insight into such a link shows that gut microbiota interact with obesity-causing diet in a way that trigger proinflammatory changes in the intestine, and together, these tilt the balance towards insulin resistance and obesity [74]. Though understanding on the molecular basis of such effects largely remains unanswered, however, initial studies point towards changed gut-permeability-mediated translocation of microbial constituents and associated upregulation of proinflammatory gene expression as one of the mechanisms that leads to changes in gut hormone production by enteroendocrine cells, modulation of gut-brain axis, and associated satiety signals. The compromise in the satiety signal predisposes to obesity where insulin resistance, type 2 diabetes, nonalcoholic hepatic steatosis, cardiovascular and neurodegenerative diseases, and several types of cancer are some of the comorbidities [75]. Toll-like receptors (TLRs) trigger and modulate the immune system. Through persistent gut bacteria antigen recognition by TLR4, a response leads to the secretion of proinflammatory cytokines. TLR4 activation in adipocytes and immunocytes significantly reduces insulin signaling, resulting in type 2 diabetes [76]. Metabolic syndrome is vastly potentiated in individuals with obesity and is defined as a group of interrelated metabolic disorders including hyperinsulinemia, hyperglycemia, hyperlipidemia, and hepatic steatosis [75]. Systemic chronic low-grade inflammation in association with obesity has a positive association with the development of metabolic syndrome based on recent studies, with chronic low-grade inflammation of the liver and adipose tissue plays a key role in the deterioration of the metabolism of the obese population. Results from our study suggested that loss of colonic mucosal expression of Jak3 led to reduced expression of mucin in colonic lumen and the development of chronic low-grade inflammation (CLGI) in the intestine of mice. Additionally, these Jak3-KO mice also showed a tendency of gaining body weight, a phenotype similar to human metabolic syndrome [1]. As previously state, loss of Jak3 expression resulted in a predisposition to DSS-induced colitis [11], and untreated mice had a tendency to gain weight. Results expressed the effects of weight gain appear to be caused by the loss of Jak3 which led to obesity and not an anomaly associated with familial transmission of microbiota from the other mice. The weight gain itself has been linked in association with IL-17a- and IL-6-mediated chronic low-grade inflammation. Jak3-KO mice also present with a statistically increased fasting blood glucose level and an epididymal fat-pad in comparison to WT mice. The livers of Jak3-KO mice also presented an increase of 130% on average in comparison to WT mice, resulting in less-dense hepatocytic nuclei to become comparatively larger. This data is consistent with early symptoms of liver steatosis. Jak3-KO mice showed exaggerated changes in comparison to WT mice when placed on a high-fat diet where 65% of the calories came from a fat source, with a high-fat diet appearing to be a promoter of metabolic syndrome in humans.

### 3.4. Jak3 in Epitheliotropic Intestinal T Cell Lymphoma (EITL)

Epitheliotropic intestinal T cell lymphoma is an aggressive primary intestinal non-Hodgkin lymphoma with a poor prognosis, and the median overall survival is approximately 7 months [77]. This intestinal lymphoma accounts for 5.4% of peripheral T cell lymphomas and 10-25% of all primary intestinal lymphomas, and the molecular alterations have not been comprehensively characterized. EITL-type I has an association with celiac disease and is more commonly found in the west, whereas type II has no known association with celiac disease and is prevalent in Asia. The mechanism of EITL works around the Jak-STAT and G-protein-coupled receptor signaling pathways, as both pathways are highly activated in cases of the disease [78, 79]. Inhibition of the Jak-STAT and GPCR pathways has shown statistically significant results in reducing the viability of primary EITL *via* targeted therapy [80]. Mutations in the Jak-STAT pathway have shown evidence in contributing to EITL with Jak3 mutations accounting for 2 out of 4 tumor samples (alterations occurring in 2 distinct activating positions: V674A and M411I). Upon further analysis, Jak3 was mutated in 14 out of 42 (33%) samples with 13 out of 42 (31%) harboring active mutations. Collectively, the Jak-STAT pathway was altered in 32 out of 42 (76%) of cases in the experiment. Jak3 can be directly targeted by small molecule inhibitors as a means of treatment for EITL.

### 3.5. Recurrent Somatic Jak3 Mutations in NK Cell Enteropathy

NK cell enteropathy or lymphomatoid gastropathy is a rare disorder that involves lymphoproliferative pathology of T and natural killer (NK) cells [81]. The involved anatomical sites can be single or multiple along the GI tract. Vagal GI symptoms include abdominal pain, constipation, and diverticulosis, as presented in patients, and lesions exhibit a chronic relapsing clinical course with no prolonged response to chemotherapy. Histological analysis reveals key clinical features that involve submucosal lamina propria with well-circumscribed but confluent infiltrate of medium-sized cells. These cells show distinct nuclear features with irregular shape, inconspicuous nucleoli, clumped chromatin, and a moderate but pale cytoplasm. Genetic analysis of the 10 patients reveals rearrangements of the gene for T cell receptor *γ* where multiple clones or a restricted pattern is observed in all 10 patients. Moreover, seven out of the 10 patients also had somatic mutations [82]. Among the mutations, 30% of the patients show identical mutations in the pseudokinase domain of Jak3 which in normal conditions provide inhibitory signal to Jak3 activation. Constitutive activation of Jak3 was further confirmed where K563-C565 deletion mutation in Jak3 leads to STAT5 phosphorylation in T-prolymphocytic leukemia. These indicate neoplastic origin of the distinct mutations in NK cell enteropathy, and therefore, lymphomatoid gastropathy can be reflected as a distinct type of neoplastic pathology. [83]

### 3.6. Remaining Questions and Future Direction

**Figure 9:**
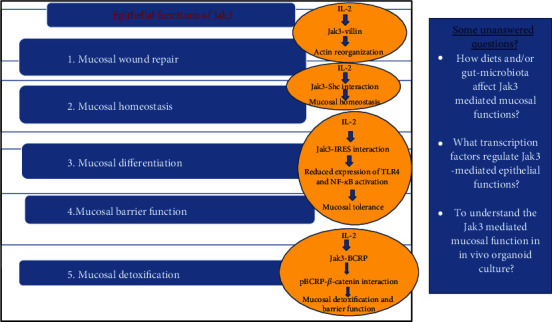
Epithelial functions of Jak3 and some unanswered questions.

Jak3 is expressed both in hematopoietic and nonhematopoietic cells where it plays significant roles. Though Jak3 has been studied thoroughly for its proinflammatory activities through immune-mediated functions however, intervention in Jak3-mediated pathways leads to several gastrointestinal complications. Therefore, an improved understanding of previously unknown mediators that coordinate mucosal functions including wound repair is important not only in understanding the biology of restitution but also in designing of novel and improved therapies to promote healing of wounds as precursor for multiple chronic inflammatory diseases. In immune cells, it is reported that IL-2 stimulation leads to tyrosine phosphorylation and activation of both Jak1 and Jak3, but IL-2 stimulates Jak3 to a significantly larger extent than JAK1 [84]. Moreover, recent report suggests an association between Jak1 rs310241 and Jak3 rs3008 polymorphisms in the risk of developing psoriasis, an epithelial abnormality [85]. Reports suggest that Jak1 interacts with Jak3, and this interaction primarily leads to the activation of STATs. However, these interactions have been noticed only in immune cells. Though the present review focuses on novel intestinal epithelial functions of Jaks in the activation of previously ignored signaling partners other than STATs, future studies should be directed towards Jak1 interactions with Jak3 and their consequences in intestinal epithelial functions that have not been reported in yet. In future, such interactions and their implication sin IEC functions should be investigated. In this review, we have demonstrated that Jak3 is a vital in regulating several critical epithelial functions including mucosal homeostasis, wound repair, barrier function, and differentiation. Moreover, as the overexpression of Jak3 also results in chronic inflammation-associated dysregulation of epithelial mesenchymal transition, which is the first step of the colon cancer, in future, it is important to decipher the molecular player and environmental factor (e.g., diet and/or gut microbiota) that regulate its expression in gastrointestinal tract (Figure 9). This is because Jak3 itself controls several other intestinal factors which are involved in multiple GI physiology. As this review demonstrated the structural component that regulates the epithelial functions through signaling molecules such as TLR4 and TNF-alpha by increasing the mucosal tolerance, understanding the transcriptional functions of Jak3 is also vital in the prognosis and treatment of intestinal diseases and associated immunological complications. Future studies need to address the transcriptional regulation of Jak3 by different transcription factors and the effect of diet in the regulation of Jak3 kinase activity. It is also important to determine the role of these responses in vivo or at least in an organoid culture, where cell survival functions may also be regulated by residential microflora that may promote cellular survival/apoptotic function-associated risk of gastrointestinal neoplasia. Future studies should also be directed to address the interactions between other three kinases, viz., Jak1, Jak2, and Tyk2 with ubiquitous (e.g., gelsolin and cofilin) or intestinal specific (e.g., villin) cytoskeletal and adherens junction proteins to develop better understanding of cytoskeletal remodeling during mucosal wound repair. Thus far, studies on the epithelial roles of Jak3 have revealed much about normal gastrointestinal function and physiology while pointing to significant potential involvement in the pathogenesis of a wide range of seemingly diverse diseases. Modulation of the Jak-mediated pathways in general and those of Jak3-mediated cellular pathways in particular, positively or negatively, regulating epithelial functions holds great promise for improved treatment of not only gastrointestinal diseases but also to so many associated health complications not to mention intestinal cancers.

## Data Availability

The data that support the findings of this study are available from the authors upon reasonable request.
